# *ina-1* expression in the somatic gonad during late larval development

**DOI:** 10.17912/W2159J

**Published:** 2016-03-01

**Authors:** Srimoyee Ghosh, Takao Inoue, Paul Sternberg

**Affiliations:** 1 Division of Biology and Biological Engineering, California Institute of Technology, Pasadena, CA; 2 Howard Hughes Medical Institute; 3 Department of Biochemistry, Yong Loo Lin School of Medicine, National University of Singapore, 117597, Singapore

**Figure 1.**
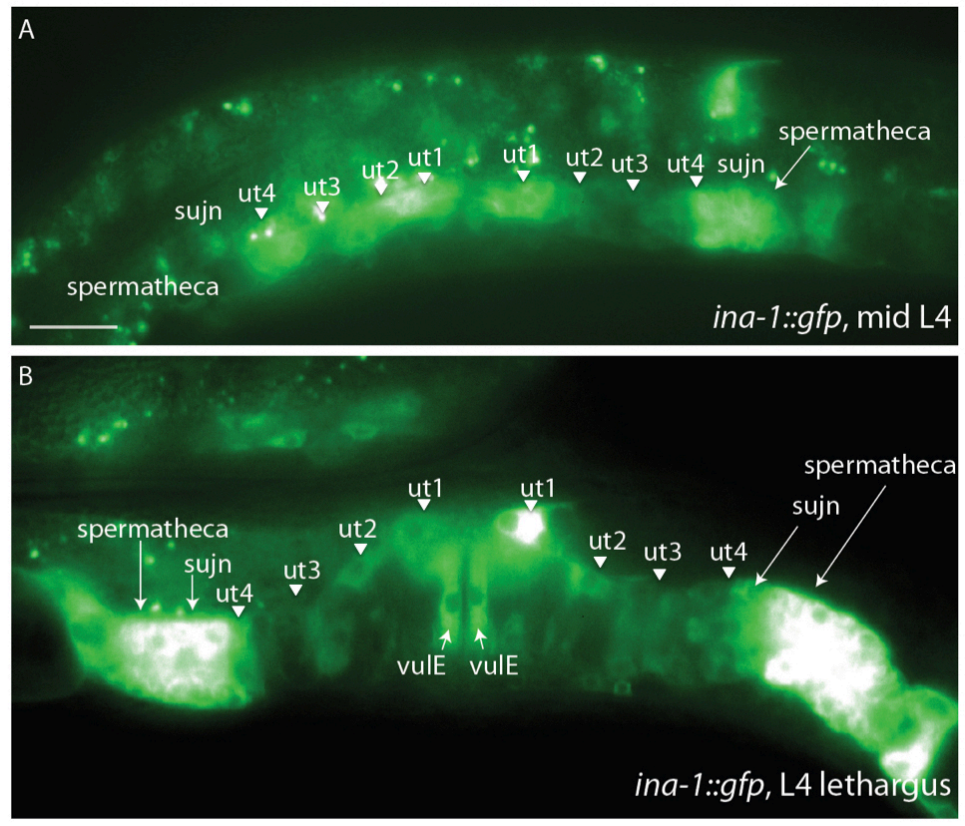


## Description

*ina-1* is expressed throughout uterine toroids. At mid L4, expression is seen in uterine toroid 1 and 4 (ut1, ut4) and in the anterior uterine toroids 2 and 3 (ut2, ut3). In addition, *ina-1*::GFP is detected in the spermathecal-uterine junction and spermatheca. At the L4 lethargus stage, bright expression is observed in uterine toroids 1 and 4, the spermathecal-uterine junction, and spermatheca. Dimmer expression is present in uterine toroid 2 and  posterior toroid 3. Fluorescence is also detected in vulE.

## Reagents

gmIs5

